# PET Imaging of Neurofibromatosis Type 1 with a Fluorine-18 Labeled Tryptophan Radiotracer

**DOI:** 10.3390/ph17060685

**Published:** 2024-05-27

**Authors:** Xuyi Yue, Erik Stauff, Shriya Boyapati, Sigrid A. Langhans, Wenqi Xu, Sokratis Makrogiannis, Uchenna J. Okorie, Azubuike M. Okorie, Vinay V. R. Kandula, Heidi H. Kecskemethy, Rahul M. Nikam, Lauren W. Averill, Thomas H. Shaffer

**Affiliations:** 1 Department of Radiology, Nemours Children’s Health, Delaware, Wilmington, DE 19803, USA; erik.stauff@nemours.org (E.S.); shriyab@seas.upenn.edu (S.B.); wenqi.xu@nemours.org (W.X.); vinay.kandula@nemours.org (V.V.R.K.); heidi.kecskemethy@nemours.org (H.H.K.); rahul_mn@outlook.com (R.M.N.); lauren.averill@nemours.org (L.W.A.); 2 Diagnostic & Research PET/MR Center, Nemours Children’s Health, Delaware, Wilmington, DE 19803, USA; sigrid.langhans@nemours.org; 3 Division of Neurology, Nemours Children’s Health, Delaware, Wilmington, DE 19803, USA; 4 Division of Physics, Engineering, Mathematics, and Computer Science, Delaware State University, Dover, DE 19901, USA; smakrogiannis@desu.edu (S.M.); ujokorie21@students.desu.edu (U.J.O.); amokorie14@students.desu.edu (A.M.O.); 5 Nemours Biomedical Research, Nemours Children’s Health, Delaware, Wilmington, DE 19803, USA; thomas.shaffer@nemours.org

**Keywords:** neurofibromatosis type 1, tryptophan metabolism, PET imaging, fluorine-18, malignant peripheral nerve sheath tumors

## Abstract

Neurofibromatosis type 1 (NF1) is a neurocutaneous disorder. Plexiform neurofibromas (PNFs) are benign tumors commonly formed in patients with NF1. PNFs have a high incidence of developing into malignant peripheral nerve sheath tumors (MPNSTs) with a 5-year survival rate of only 30%. Therefore, the accurate diagnosis and differentiation of MPNSTs from benign PNFs are critical to patient management. We studied a fluorine-18 labeled tryptophan positron emission tomography (PET) radiotracer, 1-(2-[^18^F]fluoroethyl)-L-tryptophan (L-[^18^F]FETrp), to detect NF1-associated tumors in an animal model. An ex vivo biodistribution study of L-[^18^F]FETrp showed a similar tracer distribution and kinetics between the wild-type and triple mutant mice with the highest uptake in the pancreas. Bone uptake was stable. Brain uptake was low during the 90-min uptake period. Static PET imaging at 60 min post-injection showed L-[^18^F]FETrp had a comparable tumor uptake with [^1^⁸F]fluorodeoxyglucose (FDG). However, L-[^18^F]FETrp showed a significantly higher tumor-to-brain ratio than FDG (*n* = 4, *p* < 0.05). Sixty-minute-long dynamic PET scans using the two radiotracers showed similar kidney, liver, and lung kinetics. A dysregulated tryptophan metabolism in NF1 mice was further confirmed using immunohistostaining. L-[^18^F]FETrp is warranted to further investigate differentiating malignant NF1 tumors from benign PNFs. The study may reveal the tryptophan–kynurenine pathway as a therapeutic target for treating NF1.

## 1. Introduction

Neurofibromatosis type 1 (NF1) is a complex autosomal dominant neurocutaneous disorder with an estimated incidence of 1 per 2500 births [1,2,3]. NF1 is caused by an inherited or spontaneous mutation in the NF1 tumor suppressor gene. NF1 seriously affects the quality of life in children and gradually progresses over their lifetime. Up to 80% of children develop cognitive and behavioral disorders, with 30–50% having attention deficit hyperactivity disorder (ADHD) and 40% of children having autism spectrum disorder. Children with neurofibromatosis type 1 are at a greater risk of developing plexiform neurofibromas (PNFs, benign tumors of the peripheral nerve), a known precursor lesion of malignant peripheral nerve sheath tumors (MPNSTs). In total, 8 to 13% of children with benign PNFs develop MPNSTs that behave aggressively with a high rate of local recurrence and metastasis. MPNSTs are the leading cause of mortality in NF1 patients, with a dismal overall 5-year survival rate of approximately 30% [4]. The early and accurate diagnosis of MPNSTs and the differentiation of malignant from benign tumors is critical for timely therapeutic intervention and improving prognosis. However, accurate diagnosis is extremely challenging, particularly in individuals who have multiple benign tumors. Anatomical imaging methods such as magnetic resonance imaging (MRI) and computed tomography (CT) can help identify NF1 lesions and delineate relationships with surrounding structures, but neither can reliably identify malignant degeneration [5], especially when the NF1 tumors are inhomogeneous. The major limitation of MRI and CT is their inability to monitor and confirm the malignant transformation of the lesions. Targeted chemotherapeutic agents and radiation therapies are used to evaluate therapeutic response; however, they result in complex imaging findings (such as pseudoresponse or pseudoprogression), which cannot be adequately assessed with conventional morphometric imaging techniques, further complicating accurate diagnosis. 

Positron emission tomography (PET) is a noninvasive and quantitative imaging modality to assess receptor, enzyme, and transporter levels, metabolic rate, and blood flow at the molecular level. A positron is a positively charged electron; it interacts with a free electron and turns the mass of the two particles into two 511 KeV photons emitted at almost 180 degrees to each other. After injecting into living subjects, the distribution and imaging of the photon-emitted radiotracer are acquired using back-projection methods. It utilizes positron-labeled agents with a very low mass concentration (nM to pM) to image functional changes with marginal or negligible pharmacological effects and toxicity [6]. Compared with MRI or CT, PET can detect abnormal cellular changes in the body at a very early stage. It also has advantages in distinguishing benign from malignant radiation-induced necrosis from recurrent tumors. [^1^⁸F]Fluorodeoxyglucose (FDG) is the most commonly used PET imaging agent in clinics for tumor diagnosis, staging, prognosis, and treatment evaluation [7,8]. FDG is used to detect symptomatic and asymptomatic malignant NF1 lesions in children and adolescents [9]. However, it is well known that FDG uptake is non-specific for NF1-associated tumors: high FDG accumulation has been observed in various cancer cells, inflammatory cells, and benign neoplasms [10,11]. Thus, it is difficult to differentiate the MPNSTs from benign NF1 tumors due to overlapping imaging findings, which precludes the differentiation of patients requiring biopsy or lesion excision from those who can be observed [12,13]. 

Tryptophan is an essential amino acid, and a predominant portion (~95%) of tryptophan in the human body is metabolized through the kynurenine pathway. Two rate-determining enzymes, indoleamine 2,3-dioxygenase (IDO) and tryptophan 2,3-dioxygenase 2 (TDO2), catalyze the first step in the kynurenine pathway. The dysfunction of the kynurenine pathway is implicated in many diseases, including neoplasms, epilepsy, and neuropsychiatric disorders [14,15,16,17]. The kynurenine pathway is also closely associated with malignant progression and a poor survival of patients with NF1 [18,19]. Radiolabeled tryptophan offers a novel approach for imaging NF1-associated tumors. 

To date, both preclinical research and clinical investigation of tryptophan radiotracers primarily involve carbon-11 labeled [β-^11^C]L-5-hydroxytryptophan ([^11^C]HTP) and *α*-[^11^C]methyl-L-tryptophan ([^11^C]AMT) [20,21]. However, the use of [^11^C]AMT and [^11^C]HTP is restricted to a few PET centers with access to an on-site cyclotron and radiochemistry facility due to the short half-life of carbon-11 (20 min). The rationale for using fluorine-18 labeled tryptophan is multifactorial: it is a favorable radiotracer with low background uptake, it does not require a cyclotron on site due to the relatively long half-life of fluorine-18 compared with carbon-11, and it is a metabolic radiotracer through the kynurenine pathway. To address the challenges of accurate diagnosis, monitoring, and post-treatment evaluation of MPNSTs in NF1 patients, we use a fluorine-18 labeled tryptophan PET radiotracer, 1-(2-[^18^F]fluoroethyl)-L-tryptophan (L-[^18^F]FETrp) [22], which is mainly metabolized through the unique kynurenine pathway, to identify NF1-associated neoplasms in an NF1 animal model non-invasively. 

We aim to use L-[^18^F]FETrp to detect NF1 tumors and compare the imaging results with FDG. We performed biodistribution studies and static and dynamic PET scans in NF1 mice with L-[^18^F]FETrp. We also correlated the imaging findings with a histological analysis of the tumor samples. The results showed L-[^18^F]FETrp had a comparable tumor uptake with FDG but with a low brain uptake and significantly higher tumor-to-brain ratio. Both L-[^18^F]FETrp and FDG showed high bladder uptake and were cleared through the renal–urinary pathway.

## 2. Results

### 2.1. Radiochemistry

L-[^18^F]FETrp was radiosynthesized using a one-pot, two-step strategy with decay-corrected radiochemical yields of 15–28%. The specific activity was 88–122 GBq/µmol with chemical and enantiomeric purities over 90%. The entire radiolabeling procedure took approximately 100 min. The typical dose concentration for the animal studies was 37–185 MBq/mL.

### 2.2. Biodistribution Study

**Figure 1:**
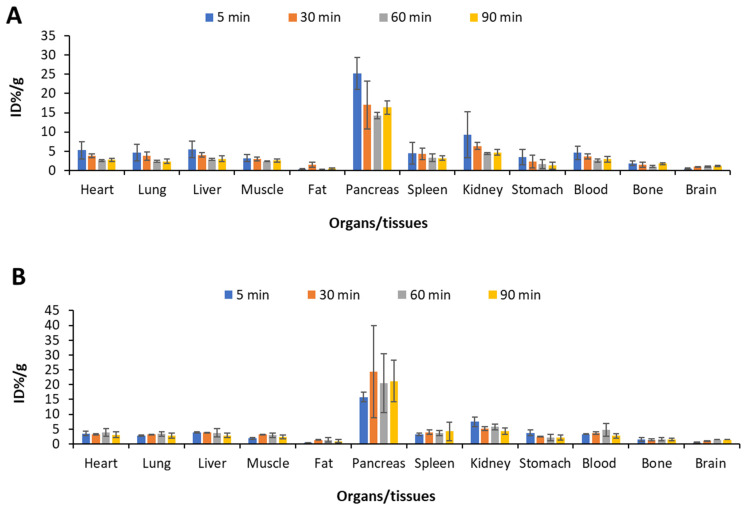
Biodistribution of L-[^18^F]FETrp at 5, 30, 60, and 90 min post-injection in wild-type (**A**) and Trp53, Nf1, and Suz12 triple mutant mice (**B**) (*n* = 3–6 per study group).

The biodistribution study of L-[^18^F]FETrp in the wild-type mice at 5 min post-injection (*n* = 4) showed the tracer had the highest uptake in the pancreas (25.1 ± 4.2 ID%/g), followed by the kidney (9.31 ± 6.01 ID%/g), liver (5.55 ± 2.15 ID%/g), and spleen (4.49 ± 2.83 ID%/g). Subsequently, there was a rapid washout of the tracer from the pancreas until 30 min compared with 90 min (17.0 ± 6.2 ID%/g at 30 min post-injection versus 16.3 ± 1.7 ID%/g at 90 min). The bone uptake was stable during the 90-min uptake period (1.91 ± 0.52 ID%/g at 5 min post-injection vs. 1.78 ± 0.24 ID%/g at 90 min), indicating the tracer was metabolically stable towards defluorination. The brain uptake was low at 5 min until 90 min post-injection (0.51 ± 0.10 ID%/g at 5 min vs.1.17 ± 0.15 ID%/g at 90 min) (Figure 1). A biodistribution study in triple mutant mice showed the highest uptake in the pancreas at 5 min post-injection (15.8 ± 1.6 ID%/g). The tracer uptake in the pancreas increased at 30 min (24.4 ± 15.5 ID%/g), followed by a decreased uptake at 60 min (20.5 ± 10.1 ID%/g). A similar pattern was observed in the spleen, muscle, and fat. Moreover, compared with the wild-type group, L-[^18^F]FETrp showed a relatively slow washout in the heart, lung, and liver, but no significant difference was observed. The bone uptake in the triple mutant group remained stable during the 90 min scans (1.49 ± 0.65 ID%/g at 5 min vs. 1.44 ± 0.45 ID%/g at 90 min), further indicating the stability of L-[^18^F]FETrp towards in vivo defluorination. 

### 2.3. MicroPET Imaging

**Figure 2:**
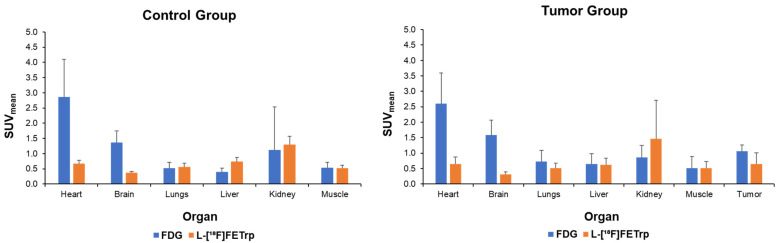
Mean SUV in the control and NF1 tumor groups at 60 min post-injection of FDG and L-[^18^F]FETrp (*n* = 4–5).

First, we performed 60-min static scans in wild-type mice and NF1 tumor mice with L-[^18^F]FETrp and compared the imaging results with the most widely used PET radiotracer, FDG. The results showed FDG had the highest uptake in the heart (standardized uptake value (SUV) 2.86 ± 1.24), followed by the brain (SUV 1.37 ± 0.38) and kidney (SUV 1.11 ± 1.41) in the control group. The FDG uptake pattern was similar in the tumor group (SUV 2.60 ± 1.00, 1.58 ± 0.49, and 0.87 ± 0.38 in the heart, brain, and kidney, respectively), with a high tumor uptake (SUV 1.07 ± 0.19) at 60 min post-injection. A relatively low FDG uptake was observed in the lung, liver, and muscle in both groups (Figure 2). In comparison, L-[^18^F]FETrp showed the highest uptake in the kidney (SUV 1.29 ± 0.27), followed by the liver (SUV 0.74 ± 0.14) and heart (SUV 0.67 ± 0.11) in the control group. Among the regions of interest, the brain had the lowest L-[^18^F]FETrp uptake (SUV 0.37 ± 0.05). In the tumor group, a similar uptake pattern with L-[^18^F]FETrp was observed in the kidney, liver, lung, heart, muscle, and brain compared with the control group. Furthermore, the brain had the lowest uptake (SUV 0.31 ± 0.08) and a high tumor uptake (SUV 0.64 ± 0.37). FDG and L-[^18^F]FETrp showed comparable muscle uptake in both groups. FDG had a higher tumor uptake than L-[^18^F]FETrp and also displayed a higher tumor-to-muscle ratio than L-[^18^F]FETrp. However, no significant difference was observed (*p* = 0.08, *n* = 4–5, *t*-test). L-[^18^F]FETrp showed a significantly higher tumor-to-brain ratio than FDG (2.0 ± 0.8 vs. 0.7 ± 0.3, *p* = 0.01), indicating a low non-specific uptake of L-[^18^F]FETrp in the mouse brains (Figure 2). 

**Figure 3:**
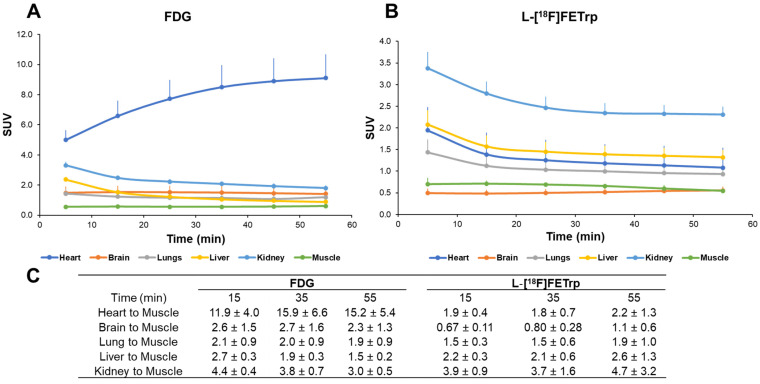
PET study of FDG and L-[^18^F]FETrp in wild-type mice. (**A**) SUV for one-hour dynamic scans of FDG in the heart, brain, lungs, liver, kidneys, and muscle (reference). (**B**) SUV for one-hour dynamic L-[^18^F]FETrp scans in the heart, brain, lungs, liver, kidneys, and muscle. (**C**) Heart, brain, lung, liver, and kidney-to-muscle ratios of FDG and L-[^18^F]FETrp at 15, 35, and 55 min post-injection of the radiotracers.

With the static scan results in hand, we focused on the kinetics of L-[^18^F]FETrp and FDG in the control and tumor groups. First, we performed 60-min in vivo microPET scans in wild-type mice with FDG and L-[^18^F]FETrp. The SUV results showed both radiotracers had a high uptake in the heart at the early time point (5.00 ± 0.65 at 5 min for FDG; 1.94 ± 0.37 at 5 min for L-[^18^F]FETrp). Then, FDG showed an increasing heart uptake during the one-hour scan period (9.11 ± 1.57 at 60 min). However, L-[^18^F]FETrp washed out slowly in the heart (1.08 ± 0.18 at 60 min). The two radiotracers showed a similar liver uptake at 5 min post-injection (2.36 ± 0.16 for FDG vs. 2.08 ± 0.33 for L-[^18^F]FETrp) and also displayed similar washout kinetics (0.89 ± 0.06 for FDG vs. 1.32 ± 0.18 for L-[^18^F]FETrp at 60 min, respectively). We used the limb muscle as a reference region and calculated both radiotracers’ organ-to-muscle ratios. The brain and heart-to-muscle ratios were significantly higher for FDG than L-[^18^F]FETrp (*n* = 4, *p* < 0.05). The results are consistent with a high non-specific FDG uptake in the brain and heart [23]. Comparable results were observed in the liver-to-muscle ratio between the two radiotracers (Figure 3).

**Figure 4:**
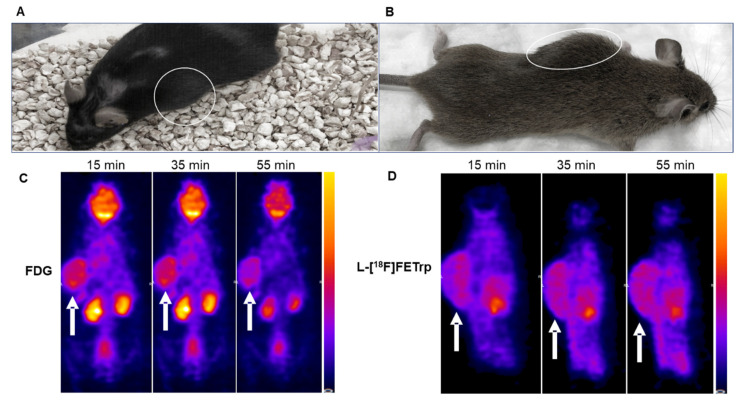
Representative PET images of FDG and L-[^18^F]FETrp in NF1 tumor mice. Pictures of two tumor mice used for FDG (**A**) and L-[^18^F]FETrp (**B**) PET scans, respectively. The tumor is indicated by white circles in (**A**,**B**). (**C**) Representative PET images of NF1 tumor at 15, 35, and 55 min post-injection of FDG (1.2 MBq). (**D**) Representative PET images of NF1 tumor at 15, 35, and 55 min post-injection of L-[^18^F]FETrp (1.9 MBq). The tumor is indicated by white arrows in (**C**,**D**).

Next, we performed PET scans with FDG and L-[^18^F]FETrp in transgenic NF1 tumor mice (Figure 4A,B). The results for representative images with NF1 tumors near the spinal cord from 15, 35, and 55 min post-injection of FDG are shown in Figure 4C. The tumor uptake was evident from 15 min until 55 min. In addition, FDG had a high accumulation in the kidneys and brain in 15 min, then decreased slowly until one hour. In contrast, L-[^18^F]FETrp also showed a high tumor uptake at 15 min post-injection, and the tumor uptake remained high at 55 min (Figure 4D). The L-[^18^F]FETrp uptake in the tumor was comparable with FDG at 15 min (SUV 1.16 ± 0.10 for L-[^18^F]FETrp vs. 1.11 ± 0.07 for FDG at 15 min). However, L-[^18^F]FETrp showed a substantially lower uptake in the brain compared with FDG (SUV 0.36 ± 0.10 for L-[^18^F]FETrp vs. 1.39 ± 0.45 for FDG at 15 min). Both the FDG and L-[^18^F]FETrp showed a high bladder uptake during the scan period (SUV 7.88 ± 4.57 for L-[^18^F]FETrp vs. 20.4 ± 8.0 for FDG at 15 min). Furthermore, no increased skull uptake was observed during the scans, indicating the stability of L-[^18^F]FETrp towards defluorination. The tumor-to-brain and tumor-to-muscle ratios were calculated for both radiotracers (*n* = 4 for each radiotracer). The results showed L-[^18^F]FETrp had a significantly higher tumor-to-brain ratio than FDG (*p* < 0.01) during the entire scans. The highest ratio was observed at the early-stage post-injection of the radiotracer (tumor/brain = 3.6 ± 1.1), then decreased until 60 min (tumor/brain = 2.4 ± 0.4). In comparison, the tumor-to-brain ratio for FDG was low and stable during the one-hour scan (0.88 ± 0.34, 1.0 ± 0.3 at 15 min and 60 min, respectively). The tumor-to-muscle ratio for L-[^18^F]FETrp was lower than FDG during the scan. However, no significant difference was observed. L-[^18^F]FETrp and FDG had a comparable tumor uptake at later times. 

### 2.4. Immunohistochemical Analysis

**Figure 5:**
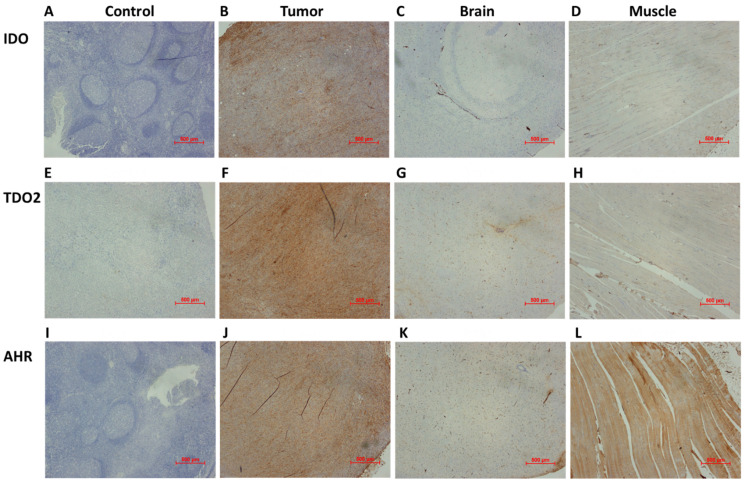
Representative immunochemistry analysis of the expression of IDO, TDO2, and AHR in control (**A**,**E**,**I**), NF1 mouse tumors (**B**,**F**,**J**), control brain (**C**,**G**,**K**), and muscle (**D**,**H**,**L**), respectively (magnification, ×4). IDO, indoleamine 2,3-dioxygenase (IDO). TDO2, tryptophan 2,3-dioxygenase 2. AHR, aryl hydrocarbon receptor.

We performed the NF1 tumor, brain, and muscle IHC analyses for IDO, TDO2, and aryl hydrocarbon receptor (AHR) (Figure 5). IDO and TDO2 are two rate-determining enzymes in the kynurenine pathway. AHR is a critical player in regulating tryptophan metabolism. Kynurenine, produced by IDO/TDO2-mediated tryptophan metabolism, is an AHR agonist. In turn, AHR regulates IDO and TDO expressions, increasing IDO and TDO2 expression in aberrant conditions [18,24]. The IHC results showed that the NF1 tumors had high IDO, TDO2, and AHR expressions, which is associated with a high tumor uptake of L-[^18^F]FETrp in PET imaging. However, both brain and muscle showed low IDO and TDO2 expressions, while the AHR expression in the muscle was high. The results may explain why the brain and muscle had a relatively low L-[^18^F]FETrp uptake compared with the tumor samples. The results further demonstrated that the high expression of enzymes in tryptophan metabolism may be positively associated with PET imaging results through the qualitative biomarker analysis. 

## 3. Discussion

The current study used a fluorine-18 label tryptophan radiotracer to image tryptophan metabolism in a neurofibromatosis type 1 animal model. The work is a continuation of our effort in the preclinical evaluation of L-[^18^F]FETrp in a medulloblastoma animal model and the translation of this imaging agent for clinical investigation [25]. NF1 is typically characterized by cutaneous and nervous lesions. The tumors are usually benign and non-cancerous. However, approximately 10% of individuals with its benign form, PNFs, still tend to develop MPNST with a dismal 5-year survival rate of 30%. This high mortality rate is due to a delayed accurate diagnosis, early metastasis, and poor response to treatment. Developing effective techniques to enhance early detection, diagnosis, and monitoring capabilities will positively impact survival. The mortality rate in patients with aggressive MPNST was over 2000-fold higher than in the general population [26,27]. Complete surgical resection with wide negative margins remains the only proven curative treatment for localized disease, with resectability depending upon the location and size, making timely and early diagnosis crucial. Furthermore, the diagnosis rendered via a biopsy may not represent the entire tumor, suggesting that upfront resection may be more valuable, further emphasizing the need for imaging biomarkers for early diagnosis before the tumor becomes unresectable or metastasizes. While anatomic imaging modalities such as MRI and CT are regularly used in clinical practice to evaluate tissue abnormalities, both show limitations in differentiating MPNSTs from their benign counterparts [9,28,29]. Therefore, it is critical to develop specific PET imaging probes for NF1 to detect functional changes early before morphological and structural abnormality occurs. Identifying biomarkers for MPNST and their validation in preclinical models are critically important to the success of clinical trials. As the most widely used PET drug in clinics, FDG has been used to detect MPNST in symptomatic and asymptomatic children and adolescents [9]. However, an overlapped SUV in benign and malignant NF1 patients was observed. Furthermore, FDG typically shows a high uptake in brains and inflammatory tissues [10,30,31], making it challenging to distinguish patients who need lesion resections from those observed cases [12,13]. 

We use tryptophan metabolism as a functional biomarker to address the clinical challenges. Tryptophan metabolism through the kynurenine pathway is modulated via the rate-limiting enzymes IDO and TDO2. Under pathological conditions, the excessive metabolism of tryptophan via the kynurenine pathway leads to changes in the kynurenine metabolic profile that have been implicated in many cancers and neurological disorders [32,33]. The carbon-11-labeled tryptophan radiotracer, *α*-[^11^C]-methyl-L-tryptophan ([^11^C]AMT), has been successfully used for the PET imaging of the kynurenine pathway in patients with epilepsy and neuro-oncology [34,35,36,37]. A recent study showed the tryptophan metabolism pathway was upregulated in human NF1 tumors and that the tryptophan metabolic rate was significantly higher in MPNSTs than in benign PNFs [18]. The research findings are the cornerstone of our hypothesis. Therefore, in the current study, we test our preliminary hypothesis that a fluorine-18 labeled PET radiotracer L-[^18^F]FETrp can non-invasively detect NF1 and show more favorable imaging properties than the widely used FDG. 

Multiple fluorine-18 labeled tryptophan analogs have been reported for imaging the kynurenine pathway or serotonin synthesis. John et al. reviewed 15 fluorine-18-labeled tracers for tryptophan metabolism. Their team compared the biological characteristics and potential for the tumor imaging of the labeled tryptophan analog by targeting the kynurenine pathway. Defluorination is one of the key limitations in determining whether the tryptophan radiotracers are suitable for clinical translation [38]. Defluorination negatively impacts imaging interpretation and quantification and can contribute to the radiotracer’s toxicity [39]. The authors conclude that L-[^18^F]FETrp is one of the two most promising radiotracers for clinical translation. L-[^18^F]FETrp is under active clinical investigation [40]. A recent first-in-human investigation of L-[^18^F]FETrp in six patients (46–56 years old) with gliomas and neuroendocrine tumors showed favorable dosimetry and demonstrated the potential clinical value of L-[^18^F]FETrp for oncological imaging [41]. The development, preclinical study, and future clinical translation of this novel ^18^F-tryptophan radiotracer show great promise to improve the management and outcomes of oncological patients, including children. 

To our knowledge, this is the first report using L-[^18^F]FETrp to image NF1 tumors. The commercially available NF1 mice carry Trp53, Nf1, and Suz12 mutations on chromosome 11 and have a high chance of developing MPNSTs. We regularly perform genotyping for the expanded cohort to identify mice with triple mutations and those susceptive to developing MPNSTs. While the kinetics of L-[^18^F]FETrp in the heart, kidney, liver, and spleen were slightly lower than in the wild-type mice, no significant difference was observed in the biodistribution study. Metabolism changes in NF1 have been reported in preclinical and clinical studies [42,43], which may explain the L-[^18^F]FETrp kinetic difference in wild-type and triple mutant mice. Both groups had a low brain uptake, unlike high and non-specific FDG uptake in brains. Negligible defluorination was observed during the 90 min, indicating the stability of L-[^18^F]FETrp towards defluorination. However, how the rate-determining enzymes in tryptophan metabolism contribute to the downstream metabolites of L-[^18^F]FETrp still needs to be determined. 

Static imaging in the control group showed the two tracers had similar uptake patterns in the lungs, liver, kidney, and muscle but significantly higher heart and brain uptake for FDG. A similar imaging profile was observed in the NF1 tumor group between the two tracers. FDG has a slightly higher tumor uptake than L-[^18^F]FETrp at 60 min post-injection but with a significantly lower tumor-to-brain ratio. Both tracers had a remarkably high bladder uptake. The hydrophilic properties of FDG and L-[^18^F]FETrp may contribute to the renal–urinary excretion route. The uptake of FDG in the heart increased during the 60-min scan, while L-[^18^F]FETrp washed out quickly. Organ-to-reference ratios showed FDG had a significantly higher brain-to-reference ratio than L-[^18^F]FETrp. In contrast, the lung, liver, and kidney-to-reference ratios were similar between the two radiotracers. Representative PET images showed both tracers had high tumor uptake at early times, then cleared out from the tumors during the one-hour scan period. However, a significant difference in the tumor-to-brain ratio was observed between FDG and L-[^18^F]FETrp, indicating the non-specific FDG uptake and low L-[^18^F]FETrp uptake in the brain. The brain uptake profile is consistent with L-[^18^F]FETrp imaging in patient-derived xenograft animal models (PDX) [44]. No significant difference was observed while the brain-to-muscle ratio was approximately two times higher for FDG during the one-hour tracer uptake compared with L-[^18^F]FETrp. We have filed an exploratory investigational new drug application (eIND) for this tryptophan radiotracer with FDA approval for clinical research in neuro-oncology patients. The preliminary study laid the foundation for extending this novel PET tracer to investigate NF1 populations and expand the patient scope in our ongoing eIND application of L-[^18^F]FETrp. 

One limitation of our study is that most Trp53, Nf1, and Suz12 mutant mice develop MPNST by approximately four months with a high mortality rate. Therefore, both L-[^18^F]FETrp and FDG image malignant NF1 tumors. The spontaneous transition from PNFs to MPNSTs in mouse models is ideal for recapitulating human PNFs to MPNSTs. However, one model that can longitudinally study benign neurofibroma formation to malignant NF1 transition is rare [45,46,47]. Our study provides a rationale that tryptophan metabolism can serve as a biomarker for NF1 tumors. More detailed studies are needed to validate whether L-[^18^F]FETrp can recapitulate PNFs to MPNSTs transition in the same or separate animal models. 

## 4. Materials and Methods

The materials and methods section describes the radiosynthesis and quality control of L-[^18^F]FETrp, the in vitro evaluation of [^18^F]FETrp in a biodistribution study, and the in vivo validation of L-[^18^F]FETrp through static and dynamic PET scans, comparing the imaging results with FDG. We also performed immunohistostaining (IHC) studies to examine the enzyme expression levels for tryptophan metabolism. 

### 4.1. Materials for Radiosynthesis of L-[^18^F]FETrp

The chemicals and supplies were purchased from vendors as reported. L-[^18^F]FETrp was purified according to published procedures [22]. In brief, a semi-preparative chiral HPLC column equipped with a chiral HPLC guard column and a short C18 column was integrated into the PETCHEM module to facilitate the purification. Quality control of L-[^18^F]FETrp was performed in an Agilent Infinity 1260 HPLC system. An Agilent ChemStation software (version C.01.07) was used for data analysis. The identity of the target radiotracer was authenticated by comparing the retention time of L-[^18^F]FETrp with the non-radiolabeled reference standard L-FETrp. D-FETrp was used to determine the enantiomeric excess value of the radiotracer. 

### 4.2. Radiolabeling Hardware and Software

L-[^18^F]FETrp was radiosynthesized in a custom-manufactured module purchased from PETCHEM Solutions Inc. (Pinckney, MI, USA). The module consists of four major components: (1) an input multiple vial positioner (MVP) for adding and drying aqueous [^18^F]fluoride solution and transferring reaction reagents during radiolabeling, (2) an output MVP for venting the reaction vessel and rinsing the reaction mixture, (3) a purification compartment consisting of a semi-preparative chiral HPLC column and a short C18 column, and (4) a formulation MVP for L-[^18^F]FETrp fraction collection and reformulation. The module features a small reaction vessel (1.5 mL) that allows flexible temperature control, multiple radioactivity detectors to monitor [^18^F]fluoride transfer and elution, a reaction vessel, and mixture loading and purification. In addition, the system includes a six-position switching valve, a 5-mL sample loop, an isocratic HPLC pump, and an ultraviolet (UV) detector. 

### 4.3. Radiosynthesis and Quality Control of L-[^18^F]FETrp

The radiosynthesis of L-[^18^F]FETrp followed the reported procedures with minor modifications [22]. In brief, [^18^F]fluoride (12–18 GBq) from a commercial source was trapped and released to a 1.5 mL reaction vessel, and the radiolabeling precursor (1–2 mg) in anhydrous acetonitrile (0.5 mL) was added to the reaction vessel containing dried [^18^F]fluoride. The mixture was heated at 100 °C for 10 min. Upon completion of [^18^F]fluoride incorporation, hydrochloric acid (aqueous solution, 2 M) in acetonitrile (0.5 mL, 1/1, *v*/*v*) was added to acidolyze the *tert*-butyloxycarbonyl and *tert*-butyl groups at 100 °C for 10 min. Then, the reaction mixture was neutralized with a basic solution (2 M NaOH, 0.25 mL) and passed through an Alumina N Sep-Pak and a C8 Sep-Pak for HPLC purification using the same mobile phase and conditions as we reported [22]. Quality control of the final radiopharmaceutical was performed in an Agilent 1260 Infinity system equipped with a Carroll-Ramsey radiation detector and an analytical chiral HPLC column. The analytical HPLC was used to determine the chemical purity, radiochemical purity, and enantiomeric excess. The isocratic mobile phase for analyzing the quality control samples consisted of 30% water and 70% USP ethanol at a flow rate of 1 mL/min. The detection wavelength was set at 230 nm. An optically pure and non-radiolabeled L-FETrp standard reference at a concentration of 1 µg/mL and a racemic mixture of L,D-FETrp at a concentration of 5 µg/mL were run in the analytical HPLC column to validate the system, according to our reported procedure [22]. The enantiomeric purity was determined using the ratio of the peak area differences to the summed areas of L- and D-[^18^F]FETrp. An enantiomeric purity of no less than 90% was used for imaging studies. The L-[^18^F]FETrp identity was authenticated by comparing the retention time between the radioactivity peak and optically pure non-radiolabeled reference standard. The retention time of the L-[^18^F]FETrp radiometric peak within ±10% compared with the retention time of the L-FETrp UV peak is considered acceptable. 

### 4.4. Biodistribution Study and PET Imaging

All animal experiments followed applicable institutional and national guidelines for the care and use of animals. The study was approved by the Nemours Institutional Committee for the Care and Use of Animals. The NF1 mice were purchased from The Jackson Laboratory (Bar Harbor, ME, USA) and bred at Nemours. The triple mutant line from the vendor carries Trp53, Nf1, Suz12 mutations on chromosome 11 and develops tumors at approximately four months, which is similar to conditions found in human NF1 patients [25,48]. Genotyping was performed to determine Trp53, Nf1, and Suz12 triple mutant and wild-type mice [49]. The following polymerase chain reaction (PCR) primers were used for the genotyping: Trp53, TGGATGGTGGTATACTCAGAGC (common), CAGCCTCTGTTCCACATACACT (mutant forward), AGGCTTAGAGGTGCAAGCTG (wild-type forward); Nf1, GGTATTGAATTGAAGCAC (wild-type forward), TTCAATACCTGCCCAAGG (common), ATTCGCCAATGACAAGAC (mutant forward); Suz12, CAGGTCTTCGAATGCTGAGTC (common), AGAGAGGGAGAAGGAGAAGCA (wild- type reverse), CTTCACATCCATGCTGAGGA (mutant reverse). For biodistribution studies, two groups were included (Trp53, Nf1, and Suz12 triple mutant group and wild-type group). The mice were injected with L-[^18^F]FETrp (0.7–2.2 MBq, 100 µL) via tail vein (*n* = 3–6 per study group) under 3–5% isoflurane in oxygen at 5, 30, 60, and 90 min post-injection, and euthanized. Heart, lung, liver, muscle, fat, pancreas, spleen, kidneys, stomach, blood, bone, and brain for both groups were harvested. All samples were weighed, and the radioactivity in these organs was assayed using an automated PerkinElmer 2480 gamma counter (Downers Grove, IL, USA) with a standard radiotracer dilution. Data were plotted to examine the biodistribution of L-[^18^F]FETrp in the peripheral organs, tissues, and brains by normalizing the body weight and injected dose. The percentage of the injected dose per gram of tissue (ID%/g) was used to calculate the radiotracer uptake. The statistical analysis was performed with a two-tailed paired Student’s *t*-test. The statistics were considered significant if the *p*-value was less than 0.05. 

Static and dynamic PET imaging in wild-type and NF1 tumor mice was performed in a PerkinElmer G4 microPET scanner. The animals were anesthetized using isoflurane in oxygen (3–5% isoflurane) five minutes before imaging. The mice were checked for lack of response to toe pinch before initiating the injection procedure; then, mice were injected with L-[^18^F]FETrp in sodium acetate solution or FDG (0.8–2.2 MBq in 100 µL) via the tail vein. The mice were immediately placed onto the temperature-controlled imaging bed under 0.5–2% isoflurane/oxygen anesthesia for imaging duration. The respiration rate was closely monitored through a built-in scanner camera to prevent animals from moving in the chamber. Dynamic PET scans were performed for one hour in list mode immediately after injection of the radiotracer. Then, the animals were euthanized before regaining consciousness from deep isoflurane anesthesia, followed by cervical dislocation. 

### 4.5. Image Reconstruction and Data Analysis

Images were reconstructed on the G4 PET scanner with defined parameters. For 60-min dynamic scans, the list mode data were binned into six frames (10 min × 6). For the PET quantification, regions of interest (ROIs), including tumor, heart, brain, lungs, liver, kidney, and muscle, were defined manually on the six 10 min frames post-injection of L-[^18^F]FETrp or FDG and applied to all other frames. ROIs were manually drawn with VivoQuant software version 4.0 (Invicro, Needham, MA, USA) following image normalization based on weight and injected dose, and PET imaging data analysis was performed using VivoQuant. The ROIs were drawn by a team member and validated by a nuclear medicine technologist to avoid any bias. The resulting quantitative data were reported in standardized uptake values (SUVs). SUVs were calculated as the ratio of regional averaged radioactivity (becquerel per cubic centimeter) to the injected radioactivity (becquerel per gram body weight).

### 4.6. Immunohistochemistry

After PET imaging, the mice were euthanized. Tumors and other organs of interest were dissected and fixed with formalin. Formalin-fixed paraffin-embedded samples were created from our core lab following standard histological processing. The samples were cut at 5 µm on a Leica RM2255 microtome (Leica, Buffalo Grove, IL, USA) and floated onto Superfrost^®^ Plus slides (Thermo Fisher Scientific, Fremont, CA, USA). The sections were heat immobilized for 60 min at 60 °C and stored at −20 °C until ready to stain. Slides were equilibrated to room temperature. The slides were then placed on the Leica Bond RX Stainer (Leica, Buffalo Grove, IL, USA), where they were dewaxed using Bond Dewax Solution (Leica, Buffalo Grove, IL, USA), then rinsed with Bond Wash Solution (Leica, Buffalo Grove, IL, USA). Heat retrieval was performed using the following conditions: ER 1 Citrate Buffer (Leica, Buffalo Grove, IL, USA) for 20 min at 100° for IDO staining, ER 2 EDTA (Leica, Buffalo Grove, IL, USA) for 20 min at 100° for TDO2 staining, ER 2-EDTA Buffer (Leica, Buffalo Grove, IL, USA) for 20 min at 100° for AHR staining. Then, the slides were stained using the Bond Polymer Refine Detection Kit (Leica, Buffalo Grove, IL, USA). The slides were incubated in their prospective antibodies for 30 min: indoleamine 2,3-dioxygenase rabbit monoclonal (1:100, Abcam, Cambridge, UK), AHR antibody mouse monoclonal (1:50, Santa Cruz, Dallas, TX, USA), antibody-TDO2, rabbit/IgG polyclonal (1:100, Proteintech, Rosemont, IL, USA), and then rinsed using Bond Wash Solution, followed by the Post Primary for 8 min and incubated in the Peroxide Block for 5 min. After additional rinses of Bond Wash Solution, the slides were developed in the 3,3′-diaminobenzidine (DAB) kits for 10 min, rinsed, and then counter-stained with hematoxylin from the kit. The slides were rinsed in deionized water, then removed from the Bond RX and placed on the Sakura Tissue-Tek^®^ Prisma™ Automated Stainer (Sakura, Torrance, CA, USA), where they were dehydrated, cleared, and then mounted in Permount^®^ (Thermo Fisher Scientific, Fremont, CA, USA).

### 4.7. Statistical Analysis 

Continuous variables were summarized by means and standard deviations (means ± SD). Biodistribution data were summarized to present the radioactivity uptake in each organ over time for the two groups: wild-type control mice and Trp53, Nf1, and Suz12 triple mutant mice. Three-dimensional ROIs were manually drawn within PET images using VivoQuant 4.0 software. PET studies included static scans in NF1 tumor mice with L-[^18^F]FETrp and FDG, dynamic PET scans in wild-type mice, and NF1 tumor mice with L-[^18^F]FETrp and FDG. The statistical analysis was performed with a two-tailed paired Student’s *t*-test with a significance level of <0.05 and a confidence level of 95%.

## 5. Conclusions

We evaluated the PET imaging of NF1 with L-[^18^F]FETrp in an animal model and compared the imaging results with commercial FDG. L-[^18^F]FETrp and FDG showed dramatically different kinetics in the hearts of wild-type mice. The NF1 tumor uptake was comparable between the two radiotracers. However, L-[^18^F]FETrp showed higher tumor-to-brain SUV ratios when compared with those of FDG. Tumor-to-muscle SUV ratios did not reveal a significant difference between tracers. L-[^18^F]FETrp was stable and showed negligible in vivo defluorination. It may serve as a promising radiotracer for guiding MPNST surgery and evaluating treatment response. L-[^18^F]FETrp may also be used for central nervous system tumors due to its low nonspecific uptake in the brain. Our animal model does not recapitulate the transition from PNFs to MPNSTs. Therefore, to further validate whether L-[^18^F]FETrp can image and differentiate NF1 tumors, a separate benign PNF animal model or an animal model with a benign to malignant NF1 transition is needed to study the capability of L-[^18^F]FETrp to image the progression of NF1-associated tumors. We also plan to perform quantitative biomarker analysis to correlate the rate-determining enzymes with the PET imaging findings and the progression of NF1 tumors. 

## Data Availability

The dataset is available upon request from the authors.
